# Biological aging processes underlying cognitive decline and neurodegenerative disease

**DOI:** 10.1172/JCI158453

**Published:** 2022-05-16

**Authors:** Mitzi M. Gonzales, Valentina R. Garbarino, Erin Pollet, Juan P. Palavicini, Dean L. Kellogg, Ellen Kraig, Miranda E. Orr

**Affiliations:** 1 Glenn Biggs Institute for Alzheimer’s and Neurodegenerative Diseases,; 2 Department of Neurology,; 3 Barshop Institute for Longevity and Aging Studies, and; 4 Department of Medicine, University of Texas Health Science Center at San Antonio, San Antonio, Texas, USA.; 5 Geriatric Research and Education Center, South Texas Veterans Health Care System, San Antonio, Texas, USA.; 6 Department of Cell Systems and Anatomy, University of Texas Health Science Center at San Antonio, San Antonio, Texas, USA.; 7 Gerontology and Geriatric Medicine, Wake Forest School of Medicine, Winston-Salem, North Carolina, USA.

## Abstract

Alzheimer’s disease and related dementias (ADRD) are among the top contributors to disability and mortality in later life. As with many chronic conditions, aging is the single most influential factor in the development of ADRD. Even among older adults who remain free of dementia throughout their lives, cognitive decline and neurodegenerative changes are appreciable with advancing age, suggesting shared pathophysiological mechanisms. In this Review, we provide an overview of changes in cognition, brain morphology, and neuropathological protein accumulation across the lifespan in humans, with complementary and mechanistic evidence from animal models. Next, we highlight selected aging processes that are differentially regulated in neurodegenerative disease, including aberrant autophagy, mitochondrial dysfunction, cellular senescence, epigenetic changes, cerebrovascular dysfunction, inflammation, and lipid dysregulation. We summarize research across clinical and translational studies to link biological aging processes to underlying ADRD pathogenesis. Targeting fundamental processes underlying biological aging may represent a yet relatively unexplored avenue to attenuate both age-related cognitive decline and ADRD. Collaboration across the fields of geroscience and neuroscience, coupled with the development of new translational animal models that more closely align with human disease processes, is necessary to advance novel therapeutic discovery in this realm.

## Introduction

By 2030, an estimated one in five Americans will be 65 years of age or older (1). As a consequence, the prevention and treatment of chronic age-related diseases are of growing public health significance (2). Alzheimer’s disease and related dementias (ADRD), which induce progressive cognitive and functional impairment, are among the top contributors to disability and mortality (3). As with many chronic conditions, aging is the greatest risk factor for the development of ADRD. After the age of 65, the incidence of ADRD nearly doubles every 5 years, and by the ninth decade of life, approximately one of every three adults meets criteria for dementia (4). Even among older adults who remain free of dementia throughout their lives, cognitive decline and neurodegenerative changes are appreciable with advancing age (5), suggesting shared pathophysiological mechanisms. Here we provide a concise overview of brain structure and function changes across the human lifespan, and mechanistic insights from translational studies highlighting biological aging processes as propagators of cognitive decline and neurodegenerative disease.

## Cognitive changes across the lifespan

As early as the third decade of life, core cognitive abilities, including processing speed, reasoning, episodic memory, and spatial visualization, begin to decline (6). Rather than a precipitous drop in old age, multivariate growth curve models have demonstrated small yet consistent diminishment in abilities across the lifespan (7). Individual cognitive domains vary with regard to their underlying neuroanatomical substrates and may decline at different rates within individuals. In aggregate, so-called “fluid skills” such as processing speed, memory, and reasoning, which rely on integration of new information, speeded response, and problem solving, tend to decrease more saliently (5). In contrast, “crystallized skills,” such as vocabulary and fund of knowledge, which are overlearned, practiced, and enhanced by experience, typically demonstrate greater stability throughout the lifespan (6). Despite variability across domains, longitudinal studies estimate that 30% to 60% of intraindividual cognitive change is attributable to a “domain-general effect” (7), which accounts for the global declines with advancing age. Similarly, experiments conducted in rodents across the lifespan have revealed age-associated deficits in late adulthood, including decrements in spatial and avoidance learning and memory (8, 9). Mice, like humans, also experience age-related changes in sensory modalities, including hearing and vision loss, which have been linked to accelerated cognitive decline (10). A recent review summarized mechanisms driving age-associated cognitive decline with a focus on changes in synaptic plasticity and intracellular calcium homeostasis (11). Other identified mechanisms entail hallmarks of aging including epigenetic changes, cellular senescence, autophagy, mitochondrial function, and inflammation, which are discussed in greater detail in later sections.

## Lifespan changes in brain morphology and function

In the absence of disease or trauma, most neurons persist throughout the lifespan, with preclinical studies suggesting that they may even outlive their host if transplanted into a longer-lived animal (12). However, in humans, cerebral gray matter volumetry gradually declines, beginning in the second decade of life, with the most appreciable changes in the frontal and parietal lobes (13, 14). Rodent models similarly indicate a reduction of gray matter volumetry in advanced age (15, 16), along with increased ventricle cerebrospinal fluid (CSF) (15) and cerebral microbleeds (16). A growing appreciation for age-associated changes in neuronal chemistry, metabolism, and morphology coincident with neuronal dysfunction and inflammation has emerged (17).

The ability to engage in new learning and memory formation, as well as other complex cognitive processes, requires coordinated action of neurons across interconnected networks. Neuronal firing patterns induce changes in synaptic plasticity that can selectively strengthen or weaken network nodes (18). In aging and neurodegenerative disease, subpopulations of neurons demonstrate reductions in intrinsic excitability, while others exude hyperexcitability, altering the signal-to-noise output (19). Aberrant hyperexcitability, in particular, has been associated with detrimental cognitive outcomes in both human and animal models (20, 21). In *Caenorhabditis elegans*, advancing age is associated with higher neuronal excitability, while dampening these changes enhances longevity (22). Exceptionally long-lived humans demonstrate upregulation of the RE1 silencing transcription factor (REST), as well as downregulation of genes implicated in excitatory transmission (22). More pronounced changes in neuronal hyperexcitability occur in the context of neurodegenerative disease, increasing seizure likelihood and accelerating cognitive decline (21). Neuropathological protein accumulation in Alzheimer’s disease (AD) disrupts the balance of inhibitory and excitatory synaptic transmission, propagating neuronal dysfunction and DNA damage (23, 24). Other changes that occur in aging and neurodegenerative disease, such as reduced mitochondrial efficiency and higher production of reactive oxygen species, have also been shown to alter glutaminergic signaling and induce hyperexcitability (25). In mouse models of AD, suppressing neuronal hyperexcitability with levetiracetam prevented synaptic loss and preserved cognitive functioning (26). A phase III clinical trial of AGB101, HOPE4MCI, is currently evaluating the efficacy of targeting hyperexcitability in adults with neurodegenerative disease (NCT03486938; ClinicalTrials.gov).

Changes in metabolites across the lifespan have further revealed new molecular targets that may provide insights into cognitive impairment, including those suggestive of altered myelination of the white matter tracts (27). Cerebral white matter is composed of lipid-rich myelin, which is essential for efficient neuronal transmission. In humans, age-related declines in white matter integrity are most pronounced in anterior brain regions and have been shown to contribute to poorer processing speed and executive function (28). In older rats, the myelin sheath increasingly splits and becomes untethered to the axon, which has been attributed to decline in structural proteins such as myelin basic protein and cyclic nucleotide phosphodiesterase (29, 30). Furthermore, the myelin-generating cells, oligodendrocytes, decline in normal aging (31), resulting in loss of myelination and age-related reductions in white matter integrity. White matter hyperintensities also become increasingly prevalent in older age (32). Histopathological studies attribute white matter hyperintensities to demyelination, gliosis, myelin parlor, and tissue rarefaction (33), which may be propagated by varied mechanisms including cerebral ischemia, neuroinflammation, and blood-brain barrier dysregulation (34, 35). In animal models, age-related reductions in white matter capillary density, coupled with atherosclerosis of the small perforating arteries, increase vulnerability to hypoperfusion and ischemia, further damaging the white matter (36, 37).

## AD neuropathological burden in aging and disease

The pathological hallmarks of AD, the accumulation of senile plaques composed of amyloid-β (Aβ) and neurofibrillary tangles derived from the aggregation of hyperphosphorylated tau, gradually accrue over decades in the context of both normal aging and neurodegenerative disease (38). With improvements in neuroimaging techniques, Aβ and transentorhinal tau have been detected in adults beginning in middle adulthood (ages 30–49; ref. 39). Evidence from AD mouse models suggests that pathological tau may spread across the brain, converting normal tau proteins into the pathological hyperphosphorylated form (40, 41). In wild-type mice, brain extracts from humans or transgenic mice with tauopathies have been shown to induce neurofibrillary tangles that can spread from the injection site to interconnected brain regions (42, 43). Aβ has also been shown to display seeding properties (44). Furthermore, Aβ and hyperphosphorylated tau, as well as broader neuropathological proteins such as α-synuclein, may interact to accelerate the overall neuropathological burden in the brain (44, 45). In Aβ-expressing mice, the addition of human tau dampens the expression of genes involved in synaptic regulation, further inducing deleterious effects on the CNS (46).

While accumulation of Aβ and tau is linked to AD, neuropathology in old age is common even in the absence of cognitive impairment. A postmortem study of 161 cognitively unimpaired adults reported that 86% displayed at least one type of neuropathology, with approximately two-thirds displaying multiple pathologies (47). Moreover, a recent meta-analysis of 4477 adults reported that approximately one-third of individuals with intermediate to high AD neuropathology remained free of dementia throughout their lives (48). Histological evidence suggests that individuals with high neuropathological burden and normal cognition may demonstrate resistance to the synaptic degradation that typically occurs with neuropathological protein accumulation (49). Several research groups are actively exploring mechanisms mediating cognitive resiliency.

## Biological aging hallmarks of cognitive decline and ADRD

**Figure 1:**
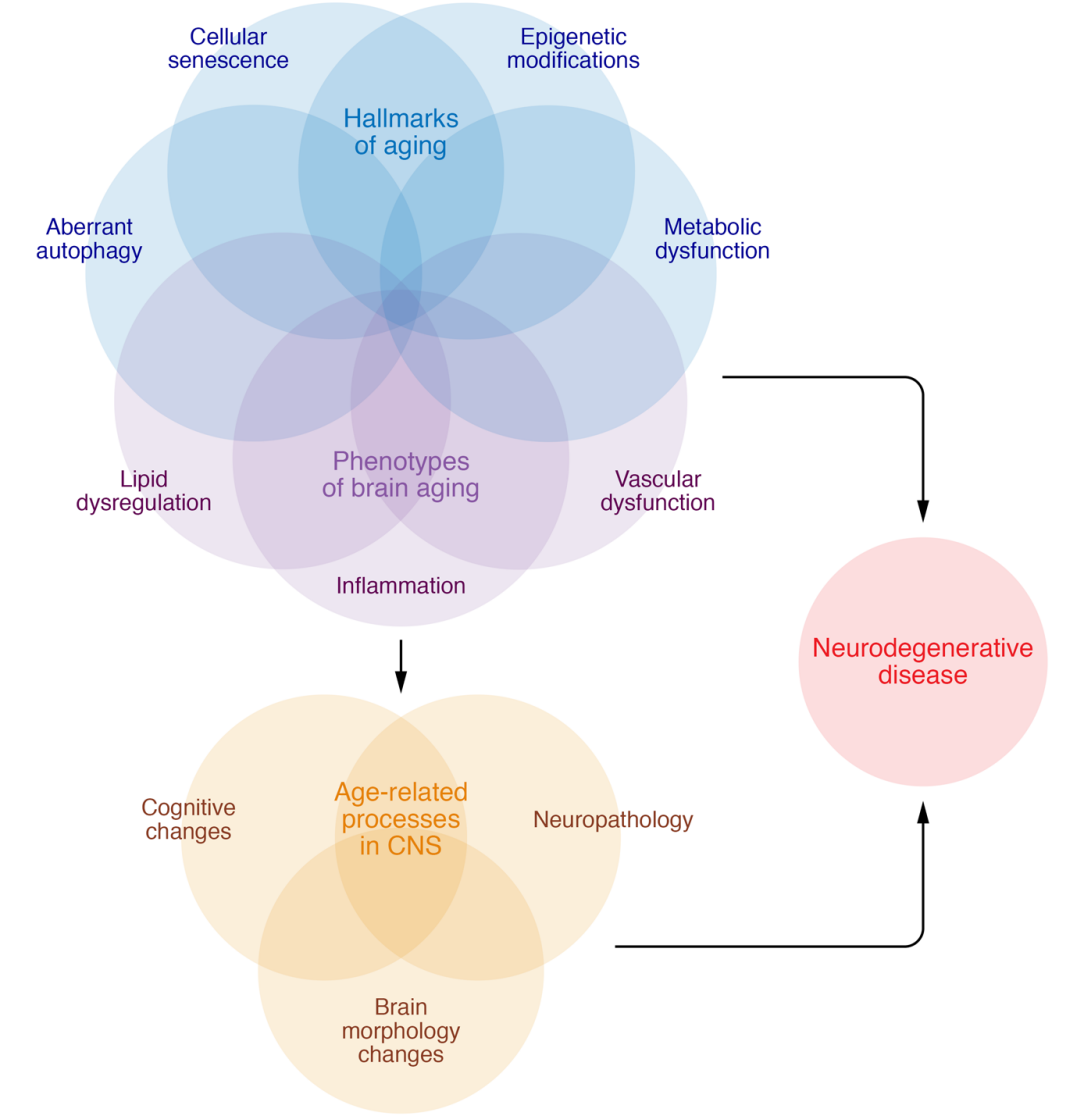
Interactions of biological aging processes with CNS changes. The hallmarks of aging, such as epigenetic modifications, cellular senescence, metabolic dysfunction, and aberrant autophagy, as well as other phenotypes of brain aging, including inflammation, vascular dysfunction and loss of blood brain barrier integrity, and lipid dysregulation, interact to contribute to age-related processes in the CNS, including cognitive decline, neuropathological protein accumulation, and brain morphology changes. These same factors are further dysregulated in neurodegenerative disease. Further investigations are necessary to determine the specific factors and sequences that force the transition between normative age-related changes and manifest neurodegenerative disease in some individuals while others remain cognitively resilient.

Population studies have demonstrated that aging is the single most influential risk factor for the development of sporadic ADRD (4). In addition, processes linked to neurodegenerative disease, including cognitive decline, cerebral atrophy, white matter degradation, and neuropathological protein accumulation, gradually manifest across the lifespan even among individuals who will remain free of dementia throughout their lives (5). Therefore, biological pathways underlying normal cognitive aging and ADRD are likely to overlap, existing along a continuum (50). Targeting fundamental processes underlying biological aging may represent a yet relatively unexplored avenue to attenuate both age-related cognitive decline and ADRD (51). The biology-of-aging field has made substantial gains in identifying the pathophysiological processes that contribute to biological aging and multisystem organ decline (52). In a seminal paper, Lopez-Otin et al. defined nine hallmarks of aging: genomic instability, telomere attrition, epigenetic alterations, loss of proteostasis, dysregulated nutrient sensing, mitochondrial dysfunction, stem cell exhaustion, altered intercellular communication, and cellular senescence (52). These aging hallmarks and others have been implicated as pathogenic factors underlying numerous chronic age-related diseases, including ADRD (Figure 1). In animal models, targeting biological aging processes has extended both lifespan and healthspan (53), suggesting the possibility that these approaches may have beneficial effects for cognitive health as well (51, 54). The following sections highlight selected aging processes that are differentially regulated in ADRD and have been mechanistically linked to pathogenesis.

### Aberrant autophagy.

The inability of postmitotic cells, such as neurons, to dilute proteotoxic burden and cellular waste through cell division increases their vulnerability to proteotoxic insults (55). Autophagy, along with the ubiquitin-proteasome system, provides relief by catabolizing proteins. Autophagy subtypes (e.g., microautophagy, chaperone-mediated autophagy, and macroautophagy) result in lysosomal degradation of substrates, including pathogenic forms of aggregate-prone proteins (i.e., Aβ, tau, and α-synuclein), lipids, dysfunctional mitochondria, and other organelles (56). Healthy neurons maintain constitutively active, highly efficient autophagy (57). Neurons in aged brains display higher levels of polyubiquitinated proteins than those in young brains; the age-associated effect becomes further elevated in the context of neurodegenerative disease (58). The requirement of autophagy activation in memory formation (59) further underscores the critical importance of its regulation for brain function. Postmortem examination of human brains with AD indicates aberrant autophagy; however, there have been conflicting reports about the directionality of dysfunction (60). Discrepancies may reflect methodological challenges associated with measuring and interpreting autophagic flux in tissue; differences in the brain regions, cell types, and species evaluated; the specific form of autophagy studied; the etiological factor(s) driving neurodegeneration; and differences in normalization controls.

Laser capture microdissection to evaluate autophagy in CA1 hippocampal neurons revealed elevated activation, but a progressive decline in lysosomal clearance across AD severity (61). Other studies indicate that Beclin-1, an autophagy-initiating protein, is reduced in AD compared with controls (62). Mechanistic studies in vitro and in vivo have demonstrated that a reduction in Beclin-1 can drive extracellular Aβ deposition (63), which protects neurons from toxic intracellular accumulation (64). Changes in Beclin-1 levels are important, as this protein negatively regulates transcription factor EB (TFEB) (65), a master transcriptional regulator of lysosome biogenesis and autophagy. Levels of nuclear (i.e., active) TFEB have been shown to progressively decrease across advancing Braak stages (66). In rodent studies, increasing TFEB reduced pathogenic tau accumulation and neurodegeneration (67); exosomal exocytosis may have contributed to the clearance of intraneuronal tau (68). Chaperone-mediated autophagy (CMA) has emerged as a critical mediator of intraneuronal tau clearance. Wild-type tau is degraded primarily through CMA; however, tau acetylation blocks CMA and redirects it toward extracellular release, increasing pathogenic spread (69–71). These studies collectively highlight the role of autophagy in eliminating intracellular neurotoxic proteins by either degrading or secreting them, as well as the essential function of extracellular clearance mechanisms for preventing the subsequent propagation of neuropathological proteins.

### Mitochondrial and metabolic dysfunction.

Mitochondria utilize oxygen for cellular respiration, extracting, transferring, and producing energy from molecular substrates derived from glucose, fat, fatty acids, and amino acids. They also contribute to calcium and iron homeostasis, cell proliferation and cell death, cell signaling, and proteostasis, thereby broadly connecting mitochondrial function with cell viability and function, and other hallmarks of aging (72). The brain is a highly metabolically active organ that requires approximately 20% of the body’s basal oxygen to optimally function (73). Reactive oxygen species (ROS) are a by-product of oxidative phosphorylation that function as a critical signaling molecule; however, their accumulation (i.e., through dysfunctional mitochondria or poor antioxidant scavenging; ref. 74) can lead to oxidative stress, lipid peroxidation, and DNA damage (75, 76). Mitochondrial changes have been proposed to drive aging (i.e., the free radical theory of aging; ref. 77) and AD (i.e., the “mitochondrial cascade” hypothesis of AD; ref. 78). The critical importance of balanced mitochondrial activity is evidenced by data demonstrating lifespan extension both by the increasing of cellular metabolism and antioxidant capacity in models (79, 80) and by interventions designed to decrease mitochondrial function or enhance ROS production (81, 82). These longevity benefits may occur through a reduction in ROS production by which improving mitochondrial oxidative stress resistance increases lifespan, suggesting that a little mitochondrial stress may be beneficial (83).

Elegant studies designed to determine the role of mitochondrial dysfunction in driving aging and disease highlight its complexity. Levels of mitochondrial DNA (mtDNA) mutations increase with age; however, results from mtDNA mutator mice indicate that these mutations do not drive oxidative stress nor accelerated aging until at extreme levels far exceeding those found in aging humans (84). The level of total mtDNA decreases with age and is reduced more in AD than in cognitively normal age-matched controls (85). Single-cell analyses indicate an increase of mtDNA deletions in AD neurons (69) that is also observed in CSF (86) and blood cells (87). Through elegant cybrid experiments (which involve transferring mtDNA from donor cells to those with identical nuclear DNA but lacking mtDNA), AD mtDNA was shown to be responsible for subtle differences in mitochondrial morphology, biogenesis, and membrane potential; oxidative stress; and calcium buffering capacity (88). The observed differences in mitochondrial phenotypes that co-occur in peripheral tissues of individuals with AD compared with controls suggest that systemic changes in mitochondrial status relevant to the brain may be identified and tracked in peripheral samples. Such data provide evidence that mitochondrial dysfunction may be upstream, and not a consequence of AD neuropathology. Nevertheless, pathogenic Aβ and tau negatively impact mitochondrial function (89, 90), which may suggest that once mitochondrial dysfunction is initiated, a pathogenic feedback loop involving oxidative stress and pathogenic protein accumulation may ensue. Further studies are needed to determine whether disease conditions (like AD) represent exacerbated “normal” age-associated changes in mitochondrial function (91) or unique divergent pathogenic processes.

### Cellular senescence.

Cellular senescence is a stress-induced cell state induced by macromolecular damage that culminates with cell cycle arrest and concomitant, often deleterious, secretory phenotype (92). Cells that become senescent evade cell death by upregulating antiapoptotic pathways and arresting the cell cycle. Senescent cells also secrete molecules including proinflammatory cytokines, chemokines, growth factors, extracellular remodeling proteins, and other signaling factors that alter the extracellular environment, collectively referred to as the senescence-associated secretory phenotype (SASP) (93). In the absence of senescent cell clearance, the SASP causes tissue damage, cell death, or the transition of other cells to become senescent, thus propagating the phenotype (94). With advancing age, senescent cells increase in tissues throughout the body, including the brain (95, 96).

Rodent studies have demonstrated senescent cell accumulation in the brain in response to accumulation of tau (90, 97) or Aβ protein (98); dysfunctional immune system (96); high-fat diet or obesity (99); insulin resistance (100); chronic unpredictable stress (101); environmental neurotoxins (102); and brain injury (103). Studies using postmortem human brain tissue have identified multiple senescent cell types in AD, including astrocytes (104), neurons (90, 105), microglia (106), oligodendrocyte precursor cells (98), and endothelial cells (107). Unbiased single-cell transcriptomics on dorsolateral prefrontal cortex from human AD revealed excitatory neurons as a prominent senescent cell type driven by *CDKN2D* (encoding p19) that overlapped with neurons bearing neurofibrillary tangles (NFTs) (105). In contrast, bioinformatics analyses of data derived from bulk tissue from healthy human tissue donors revealed that prominent senescent cell types in the brain included endothelial cells and microglia driven by *CDKN1A* (108). These studies, both conducted by our group, highlight potential differences in senescent cell types (a) in health versus disease; (b) possibly as a reflection of the starting material (i.e., single-cell, single-nucleus, or bulk tissue analyses); and (c) owing to differences in the predetermined criteria for senescence. Immunosenescence, described below, drives senescent cell accumulation in the brain (96). Microglia, the macrophage-like cells of the brain, clear NFT-bearing neurons that display phosphatidylserine on their surface (109). Given that microglia become senescent and dysfunctional after clearing these possibly senescent, NFT-bearing neurons (105), therapeutic strategies to help remove senescent cells from the brain may alleviate senescent cell burden, inflammation, and disease propagation (90, 98). Clinical trials are currently under way to test this approach (110, 111).

### Epigenetic changes.

Epigenetic processes allow cells to integrate external stimuli into their genome to impact gene expression without altering the DNA sequence. These dynamic, reversible modifications include DNA methylation, chromatin remodeling, histone modification, and noncoding RNA regulation (microRNAs) (112). Neuronal epigenetic changes are crucial for synaptic plasticity and new memory formation (113). With age, DNA methylation in the brain trends toward global decreases, but there are sex-dependent dimorphisms (114, 115). Given that DNA methylation inhibits gene transcription, these changes may result in elevated gene expression. Genes implicated in AD, including those coding for *APP*, *MAPT*, *BDNF*, *ABCA7*, *ANK1*, *BIB1*, *SORL1*, and *SIRT1*, show differential methylation between individuals with AD and controls (116, 117). Breast cancer type 1 susceptibility protein (BRCA1), a DNA repair protein typically associated with breast cancer, is hypomethylated in AD. Elevated BRCA1 localizes to the cytosol, where it coaggregates with insoluble tau. In vitro studies suggest that this impacts neurite and dendritic spine morphology (118). Moreover, epigenetic age acceleration was found to be heritable in AD, where it was associated with neuropathological protein accumulation and cognitive decline (114, 119). Collectively these data suggest that epigenetic changes may increase AD susceptibility.

The frequency and pattern of epigenetic changes, specifically DNA methylation at CpG sites, can be used to generate an algorithm for comparing chronological age with biological age, termed an epigenetic clock. There are currently more than seven different epigenetic clocks developed for human assessments (120–126) and others for mouse models (127, 128). These differ in numbers of methylated CpGs, tissue type, and study populations. The current clocks lack correlation among them (129, 130). Nevertheless, understanding the relationships between DNA methylation, age, longevity, and age-related disease may hold promise to predict disease, including diseases relevant to the brain (131). While initial epigenetic clocks were based in blood, recent advances are moving to the brain to predict cortical age (130, 131). The recently developed Cortical clock provides evidence supporting the use of the epigenome to inform regarding brain aging and pathologies (132). The Cortical clock was trained using postmortem cortical tissue from older adults, which tracked better with AD diagnosis and Aβ deposition than clocks trained using blood. While blood-based clocks correlated with chronological age at death when applied to cortical tissue (130), only the Cortical clock significantly associated with tau and Lewy body pathology, highlighting the importance of considering tissue-specific epigenetic changes in these predictions.

Chromatin remodeling and chromatin heterogeneity (or what has been termed epigenetic noise) also increase with age. Histone acetylation tends to decrease with aging, resulting in a more condensed chromatin structure and consequent transcriptional changes (133). A recent assessment of postmortem human brain tissue revealed an upregulation of two histone acetyltransferases, H3K27ac and H3K9ac, that were linked with Aβ pathology and neurodegeneration by human proteomics data and a transgenic fly model (134, 135). Three AD mouse models and one nonhuman primate model displayed epigenetic changes that differed across models (136). This work again emphasizes the complexity of genetic and epigenetic influence on disease progression, as well the importance of matching model systems to the underlying pathogenic process in question.

Unlike the above-mentioned epigenetic alterations, microRNAs (miRNAs) influence gene expression post-transcriptionally by binding to mRNA (137). miRNAs play critical roles in AD pathology, including modulating Aβ and tau production/function, synaptic plasticity, neuronal growth, apoptosis, and inflammatory response (138). In AD, disruptions have been noted in several miRNAs, including miRNAs 9, 124, 125b, 132, 146a, and 155, which may have the potential to serve as both biomarkers and therapeutic agents (138, 139).

### Vascular dysfunction and diminished blood-brain barrier integrity.

Epidemiological evidence supports an association between risk factors for cardiovascular disease, cerebrovascular dysfunction, and cognitive impairment. More than 50% of individuals with ADRD have concomitant vascular pathologies that increase with advancing age (140). Furthermore, growing evidence indicates that the molecular mechanisms associated with both vascular and ADRD pathologies act synergistically to compromise cognition (140). Vascular contributions to cognitive impairment and dementia (VCID) derive from age-related changes to the neurovascular unit (NVU), which is composed of nonfenestrated endothelial cells, pericytes, smooth muscle cells, astrocytes, microglia, oligodendroglia, and neurons (141). The NVU facilitates normal brain function by ensuring neurovascular coupling, the physiological mechanism whereby cerebral blood flow is matched to neuronal metabolic demands (142). With aging, and to a greater extent in neurodegenerative disease, there is a loss of pericytes, which has been associated with diminished cerebral blood flow delivery in both human and animal models (143). In mouse models of AD, pericyte loss has also been shown to reduce Aβ clearance, further propagating neuropathological protein accumulation (144). In addition, age-related changes in mitochondrial efficiency and the upregulation of ROS induce endothelial dysfunction, which diminishes the bioavailability of the vasodilator nitric oxide and further dampens neurovascular coupling (145).

The NVU is also important for the maintenance of the blood-brain barrier (BBB), which controls transport of substances across the endothelium into the CNS through specific transporters on both the luminal and abluminal surfaces (141, 146). BBB integrity declines in normal aging and even more dramatically in ADRD. Loss of BBB function induces capillary leakage, brain leukocyte infiltration (141), ingress of toxic substances, and upregulation of TGF-α signaling in astrocytes, resulting in disruption of the brain milieu and neuronal dysfunction (147). BBB leakage has been identified in the hippocampi of individuals with mild cognitive impairment, which correlates with CSF levels of PDGF-β, a marker of damaged pericytes (146). Loss of BBB integrity further drives neuroinflammation, which has been implicated in aging and ADRD.

### Inflammaging.

It has been well established that systemic inflammation increases with age, as evidenced by higher circulating levels of proinflammatory cytokines (i.e., IL-1β, IL-6, TNF-α) and immune dysregulation (loss of vaccine efficacy, increased morbidity upon infection, rises in cancer incidence, and enhanced autoimmunity). This “inflammaging,” a term originally coined by Claudio Franceschi, is thought to contribute to systemic pathologies that develop with age, including ADRD (148, 149). Numerous studies have shown correlations between circulating proinflammatory mediators and progression of neurodegenerative diseases, suggesting that *peripheral* inflammation contributes to the development of chronic brain inflammation (150–154). In addition, recent studies using CSF to interrogate neuroinflammation directly in the CNS have shown mixed results. For example, in adults without measurable cognitive impairment, increased cytokine levels in the CSF were, surprisingly, associated with lower tau and Aβ levels (155). In addition, higher plasma levels of IL-12p70 and IFN-γ have been associated with protection against cognitive decline in cognitively unimpaired adults (156). Thus, it is possible that mild neuronal inflammation may provide some early protection. On the other hand, as disease etiology progresses, an association with neuroinflammatory markers, including C-reactive protein (CRP), triggering receptor expressed on myeloid cells 2 (TREM2), intercellular adhesion molecule 1 (ICAM1), IFNs, and the IL-1 family, is typically reported (157–160).

Aging elicits pleiotropic outcomes, reflecting many different factors that contribute to increased neuroinflammation; these have been extensively reviewed (161–163) and will be only briefly mentioned here. For example, brain microglia, analogous to systemic macrophages, become activated by tissue damage or pathogens and release proinflammatory mediators (reviewed in ref. 164). Inflammation can also alter Aβ clearance through effects on the NLRP3 inflammasome (165). Age-associated changes in the cells of the adaptive immune system may contribute as well. For example, the proportion of CD4^+^ T cells that are phenotypically suppressive, designated Tregs (expressing FOXP3), increases with age. Tregs have been shown to play both protective and pathogenic roles in neurodegenerative diseases (166, 167). Indeed, in a mouse model of AD, transient inactivation of Tregs showed improved cognition and decreased inflammation (168). T cells may also play a more direct role in neurodegenerative disease through recognition of their cognate antigen(s) through the cell surface T cell receptor (TCR) as is seen in multiple sclerosis, an autoimmune disorder in which pathogenic T cells recognizing myelin peptides damage the tissue. In pilot Aβ vaccination studies for AD, there was an induction of neuroinflammation, which in some cases led to a devastating meningoencephalitis due to proinflammatory CD4^+^ T cells (169). Even without immunization, autoimmune responses to neuronal peptides could develop, and in that case, one might expect to find a more restricted TCR repertoire due to selection of those antigen-specific T cells in the CNS. Indeed, this has recently been reported for CD4^+^ T cells in the CSF of individuals with AD (170). However, it is not clear whether the T cell clonotypes responding are “helper” T cells (CD4^+^FOXP3^–^) or “suppressive” Tregs (CD4^+^FOXP3^+^), which could be either pathogenic or protective.

### Lipid dysregulation.

Genetic linkage, large-scale genome-wide association, and exome sequencing studies have also repeatedly linked lipid metabolism–related genes/loci and rare variants with AD, including apolipoprotein E (*APOE*), *CLU*, *ABCA7*, *SORL1*, *TREM2*, *PICALM*, *INPP5D*, and *PLCG2* (reviewed in refs. 171–173). Several lipid-related gene variants, including *APOE*, have also been associated with human longevity (174). The first longevity-assurance gene (*LAG1*) discovered in yeast was found to code for a ceramide synthase (175). Ceramides comprise a class of lipids that play essential roles both as intermediates in the biosynthesis of more complex sphingolipids, and as signaling molecules that participate in a plethora of biological processes (176), including apoptosis, inflammation, insulin signaling, mitochondria function, cellular senescence, telomerase activity, and autophagy (177, 178).

Alterations in brain lipid composition occur in both normal aging and neurodegenerative disease. The brain is the richest organ in terms of lipid content and diversity, largely owing to the abundance of lipid-rich myelin (179). Lipidomics, the large-scale study of pathways and networks of cellular lipids in biological systems, has revealed specific lipid profiles associated with AD (180, 181) and aging (182). For example, early accumulation of ceramide levels in the AD brain has been consistently reported by multiple groups (183, 184). On the other hand, sulfatides, a class of sulfoglycolipids highly enriched in myelin, have been reported to be specifically and dramatically reduced at the earliest clinically recognizable stages of AD (185–187). Brain sulfatide levels in patients with AD and in animal models strongly correlate with the onset and severity of Aβ deposition (188, 189). Mechanistic studies in animal models have revealed that sulfatide deficiency in AD occurs in an isoform-specific manner (190, 191) and that sulfatide losses are sufficient to induce AD-like neuroinflammation and cognitive decline (192). Moreover, levels of the phospholipid plasmalogen have been consistently shown to decline not only in the brains of individuals with AD, but also in circulation, with ethanolamine plasmalogen deficits closely associating with disease severity (193). Notably, human brain plasmalogen levels have also been reported to decline with normal aging, decreasing dramatically by around 70 years of age (194).

## Conclusions

Chronological aging is accompanied by molecular, cellular, and systems-level processes with underlying biology that may modulate susceptibility to neurodegenerative disease (50, 51, 195). Applying current insights from the biology-of-aging field to age-associated neurodegenerative diseases offers an opportunity to explore and target new cellular and molecular processes. We have focused on a few selected hallmarks of aging for which interventions are moving to clinical trials in the context of mild cognitive impairment/early AD. Though still an emerging field, geroscience-motivated approaches are appealing for the treatment of complex age-associated diseases, like AD. The synergistic interactions across biology-of-aging pathways raise optimism that effective targeting of one may exert broader beneficial influences (51, 196). As highlighted above, the transition in these cellular and molecular processes over the course of the disease is complex and may be nonlinear. Early upregulation of specific processes, such as cellular respiration and senescence, may help mitigate neurodegenerative disease changes; however, these same processes may be detrimental over time by perpetuating oxidative stress and inflammation (197). Early trials exploring geroscience-motivated approaches for the treatment of AD will provide critical information on this strategy. For example, NCT04685590, led by our team, will focus on geroscience outcomes as well as AD biomarkers and cognitive changes. Other studies are targeting mitochondrial function with NAD^+^ precursors (198) (NCT04078178, NCT04430517) and nutrient sensing and handling with rapamycin (NCT04200911, NCT04629495). As these early trials are under way, advances in the basic biology of aging are needed to continue shedding light on cell type specificity and interactions across biology-of-aging hallmarks, and to refine model systems through efforts including Model Organisms Development and Evaluation for Late-Onset Alzheimer’s Disease (MODEL-AD) (199). Furthermore, cross-disciplinary training and collaboration across the fields of neuroscience and geroscience will be crucial for advancing treatments that target age-related dysfunction across systems in an effort to optimize both physical and cognitive functioning throughout the lifespan (200).

## Version 1 05/16/2022

Electronic publication
